# Rapid-Onset Obesity with Hypothalamic Dysfunction, Hypoventilation, and Autonomic Dysregulation (ROHHAD): exome sequencing of trios, monozygotic twins and tumours

**DOI:** 10.1186/s13023-015-0314-x

**Published:** 2015-08-25

**Authors:** Sarah F. Barclay, Casey M. Rand, Lauren A. Borch, Lisa Nguyen, Paul A. Gray, William T. Gibson, Richard J. A. Wilson, Paul M. K. Gordon, Zaw Aung, Elizabeth M. Berry-Kravis, Diego Ize-Ludlow, Debra E. Weese-Mayer, N. Torben Bech-Hansen

**Affiliations:** Department of Medical Genetics, Cumming School of Medicine, Alberta Children’s Hospital Research Institute, University of Calgary, Calgary, AB Canada; Center for Autonomic Medicine in Pediatrics (CAMP) in Department of Pediatrics, Ann & Robert H. Lurie Children’s Hospital of Chicago and Stanley Manne Children’s Research Institute, Chicago, IL USA; Department of Anatomy and Neurobiology, Washington University of Medicine, St Louis, MO USA; Department of Medical Genetics, University of British Columbia and Child & Family Research Institute, Vancouver, B.C. Canada; Department of Physiology and Pharmacology, Alberta Children’s Hospital Research Institute and Hotchkiss Brain Institute, Cumming School of Medicine, University of Calgary, Calgary, AB Canada; Departments of Pediatrics, Neurological Sciences, and Biochemistry, Rush University Medical Center, Chicago, IL USA; Department of Pediatrics, Division of Pediatric Endocrinology, University of Illinois at Chicago, Chicago, IL USA; Northwestern University Feinberg School of Medicine, Chicago, IL USA

**Keywords:** ROHHAD, Obesity, Hypothalamic dysfunction, Autonomic dysregulation, Hypoventilation, Genomics, Genetics, Exome sequencing, Next-generation sequencing

## Abstract

**Background:**

Rapid-onset Obesity with Hypothalamic Dysfunction, Hypoventilation, and Autonomic Dysregulation (ROHHAD) is thought to be a genetic disease caused by *de novo* mutations, though causative mutations have yet to be identified. We searched for *de novo* coding mutations among a carefully-diagnosed and clinically homogeneous cohort of 35 ROHHAD patients.

**Methods:**

We sequenced the exomes of seven ROHHAD trios, plus tumours from four of these patients and the unaffected monozygotic (MZ) twin of one (discovery cohort), to identify constitutional and somatic *de novo* sequence variants. We further analyzed this exome data to search for candidate genes under autosomal dominant and recessive models, and to identify structural variations. Candidate genes were tested by exome or Sanger sequencing in a replication cohort of 28 ROHHAD singletons.

**Results:**

The analysis of the trio-based exomes found 13 *de novo* variants. However, no two patients had *de novo* variants in the same gene, and additional patient exomes and mutation analysis in the replication cohort did not provide strong genetic evidence to implicate any of these sequence variants in ROHHAD. Somatic comparisons revealed no coding differences between any blood and tumour samples, or between the two discordant MZ twins. Neither autosomal dominant nor recessive analysis yielded candidate genes for ROHHAD, and we did not identify any potentially causative structural variations.

**Conclusions:**

Clinical exome sequencing is highly unlikely to be a useful diagnostic test in patients with true ROHHAD. As ROHHAD has a high risk for fatality if not properly managed, it remains imperative to expand the search for non-exomic genetic risk factors, as well as to investigate other possible mechanisms of disease. In so doing, we will be able to confirm objectively the ROHHAD diagnosis and to contribute to our understanding of obesity, respiratory control, hypothalamic function, and autonomic regulation.

**Electronic supplementary material:**

The online version of this article (doi:10.1186/s13023-015-0314-x) contains supplementary material, which is available to authorized users.

## Background

Rapid-onset Obesity with Hypothalamic Dysfunction, Hypoventilation, and Autonomic Dysregulation (ROHHAD) is a complex and devastating disease whose etiology is poorly understood, despite its initial description 50 years ago [1]. The condition, previously termed “late-onset central hypoventilation syndrome with hypothalamic dysfunction” is extremely rare, with fewer than 100 cases reported in the literature, and occurs sporadically, with no clear-cut family history for inheritance of the phenotype [1–7]. In addition to the hypothalamic, respiratory and autonomic manifestations that are hallmarks of the disease, about 40 % of ROHHAD patients develop benign tumours of neural crest origin [2–4]. Because of the heralding feature of the rapid-onset obesity (20–30 lb over a 3–6 month period in, typically, a 2–7 year old, otherwise healthy, child), affected children should come to the attention of their pediatricians early in the clinical course. However, due to the variable timing and onset of other features, coupled with the presumption of exogenous obesity, a ROHHAD diagnosis is often delayed or missed, potentially leading to fatal central hypoventilation, cardiorespiratory arrest, and impaired neurocognitive development. Conversely, many children with marked and potentially rapid weight gain, who do not meet the additional clinical criteria for ROHHAD, are inappropriately labeled as having ROHHAD. While diagnostic criteria and knowledge of the disease course have improved since the introduction of the ROHHAD acronym by Ize-Ludlow and coworkers in 2007 [2], these issues remain a challenge. Thus, unambiguous diagnosis of ROHHAD is difficult but essential – not only for scientific inquiry but also for appropriate patient care. The identification of a diagnostic marker (genetic or otherwise) has the potential to decrease morbidity and mortality of patients with ROHHAD, and to guide future intervention and research on this disease.

As many ROHHAD features are reminiscent of other neurocristopathies of genetic origin, such as Congenital Central Hypoventilation Syndrome (CCHS) [7], a genetic basis to ROHHAD has been hypothesized. The sporadic appearance of a syndrome with tumour predisposition and an apparently primary disturbance of body growth is consistent with somatic mosaicism or constitutional inheritance of a *de novo* dominant mutation (as seen in Sotos syndrome [8], Proteus syndrome [9], CLOVES syndrome [10], and Weaver syndrome [11]), though epigenetic mechanisms could also be postulated, as have been shown to occur in Beckwith-Wiedemann syndrome [12]. However, previous candidate gene studies in ROHHAD using Sanger sequencing have not revealed a clear candidate gene that can account for the full ROHHAD phenotype [2, 4, 13].

To date, most known disease-causing mutations have been identified within the coding portion of the genome. Whole exome sequencing (WES) among small, carefully-phenotyped cohorts has proven to be a very effective means of identifying the cause of rare pediatric diseases, a fact well demonstrated in a large national exome sequencing effort (FORGE Canada Consortium) that found disease-causing variants for 146 rare pediatric diseases, including variants in 67 genes not previously linked to any human disease [14]. Thus, WES is a highly cost-effective and efficient method for solving the genetic basis of rare diseases. To search for both constitutional and somatic *de novo* sequence variants that could be the cause of this severe pediatric disease, we used WES to analyze seven ROHHAD trios, the unaffected monozygotic (MZ) twin of one of these seven patients, and tumours isolated from four of these seven patients as a discovery cohort. We also assembled a replication cohort of 28 singleton ROHHAD cases in which to analyze candidate genes identified through WES.

## Methods

### Criteria for preliminary diagnosis

The basic criteria for consideration of the diagnosis of ROHHAD were published in Ize-Ludlow et al. (2007) [2]. Briefly, features included: 1) onset of rapid and extreme weight gain after age 1.5 years (typically 2–7 years) in a previously non-obese and seemingly normal child, 2) evidence of hypothalamic dysfunction, 3) alveolar hypoventilation, and 4) features of autonomic dysregulation.

### Patient selection

The Center for Autonomic Medicine in Pediatrics (CAMP) at Ann & Robert H. Lurie Children’s Hospital of Chicago and the Stanley Manne Children’s Research Institute is a Center of Excellence for the study of ROHHAD. Medical records for each proband referred to CAMP were reviewed to confirm ROHHAD characteristics. Cases were then tested and confirmed negative for any CCHS-related *PHOX2B* gene mutations. All of the patients selected for exome sequencing were evaluated clinically in the CAMP laboratory for serial characterization of the ROHHAD phenotype (control of breathing, hypothalamic dysfunction, and autonomic dysregulation). Patients who met strict diagnostic criteria for ROHHAD were offered inclusion into both the International ROHHAD REDCap Registry and the ROHHAD Genetic Inquiry project (both IRB-approved). In this cohort, all patients demonstrated rapid-onset obesity, hypothalamic dysfunction, hypoventilation requiring artificial ventilation, and autonomic dysregulation. All (35) patients who consented to the Genetic Inquiry project, provided a peripheral blood sample and, where appropriate, provided a frozen neural crest tumour tissue sample, were included in this study. All (7) patients whose parents also consented to the Genetic Inquiry project and provided a peripheral blood sample were included in the *discovery cohort* for trio analysis. The other 28 participants formed the *replication cohort*, of which exome sequence data were obtained for 9 and the remaining 19 were analyzed by Sanger sequencing, only in genes of interest.

### Ethics, consent and permissions

This study was approved by the Ann & Robert H. Lurie Children’s Hospital of Chicago IRB (study ID: 2009–13904) and the University of Calgary Conjoint Health Research Ethics Board (study ID: REB13-0164_REN2). All participants provided informed consent to participate, including consent to publish the data herein.

### Sample collection and DNA extraction

Genomic DNA was isolated from blood and tumour samples, using a Puregene reagent kit (Qiagen). Tumours included both ganglioneuroma and ganglioneuroblastoma samples (Table 1).

**Table 1:** Phenotype of probands with Rapid-onset Obesity with Hypothalamic Dysfunction, Hypoventilation, and Autonomic Dysregulation (ROHHAD) in discovery and replication cohorts

Cohort	Number of probands	Gender	Race/ethnicity	Age at rapid-onset obesity onset mean (range) in years	Hypothalamic dysfunction	Hypoventilation	Artificial ventilation	Autonomic dysregulation	Tumour of neural crest origin^a^
Discovery Cohort (Trios)	7	5 F; 2 M	7 Caucasian	4.4 (2–8)	7/7	7/7	7/7	7/7	5/7
Replication Cohort	28	16 F; 12 M	17 Caucasian; 5 Hispanic; 6 Asian	3.7 (1.8-8)	28/28	28/28	28/28	28/28	10/28

^a^Tumors were ganglioneuromas or ganglioneuroblastomas of the chest and abdomen

### Whole exome sequencing and analysis

Exome captures were completed using the Agilent SureSelect V5 + UTRs capture kit. Massively parallel sequencing was performed on a SOLiD platform, at the Alberta Children’s Hospital Research Institute (ACHRI), and sequences were aligned to the human reference genome (GRCh37) using Lifescope Genomic Analysis Software 2.5 (Life Technologies), (see Table 2). Variants were called using DeNovoGear [15] for the trio, twin, and tumour analyses; and the Genome Analysis Toolkit (GATK) Haplotype Caller (Version 3.3) [16] for the recessive and dominant analyses; and annotated for filtration and prioritization using ANNOVAR [17]. For the trio analysis, candidate variants were identified as novel or rare (MAF < 0.005, according to 1000 Genomes Project [18], the Exome Variant Server [EVS; [19]] and the Exome Aggregation Consortium [ExAC; [20]]) exonic, UTR, or splice site (within 2 bp of an exon) variants not within a segmental duplication. Candidate variants were further filtered to exclude UTR variants for the recessive analysis, and both UTR and synonymous variants for the dominant analysis. Candidate variants were assessed using Combined Annotation Dependent Depletion (CADD) [21] and PolyPhen-2 [22] (for non-synonymous variants) to predict deleteriousness. CADD scores are phred-scaled, so a score of 10 indicates the pathogenicity score of a variant is in the top 10 % of the scores for all possible human variants; a score of 20 indicates it is in the top 1 % etc. PolyPhen-2 scores represent the probability that the variant is deleterious.

**Table 2:** Details of exome sequencing

Cohort	Sequencing site^a^ (Capture Kit, Sequencing platform, Aligner)	Participant ID	Description	Mean depth of coverage	Mean depth of coverage, cohort average (SD)	% of target region covered at least 20×	% of target region covered at least 20×, cohort average (SD)
Discovery cohort	ACHRI (Agilent SureSelect V5 + UTRs, Life Technologies SOLiD 5500xl, LifeScope 2.5)	18	Proband	141.4	132.7 (14.1)	91.88	931.79 (0.81)
		31	Mother of 18	143.9		91.62	
		22	Father of 18	136		90.93	
		37	Monozygotic twin of 18	133.5		90.97	
		25	Proband	125.4		92.01	
		51	Mother of 25	149.6		92.81	
		50	Father of 25	151.2		92.84	
		27	Proband	139.2		91.87	
		32	Mother of 27	128		91.11	
		33	Father of 27	126.3		90.94	
		41	Proband	124.5		90.92	
		47	Mother of 41	142.1		92.97	
		46	Father of 41	139.4		92.65	
		42	Proband	133.4		92.04	
		43	Mother of 42	127.2		91.55	
		44	Father of 42	119.1		90.91	
		45	Proband	122.2		91.73	
		48	Mother of 45	136.3		92.43	
		49	Father of 45	165.1		93.58	
		57	Proband	124.7		91.45	
		59	Mother of 57	113.4		90.55	
		58	Father of 57	98.5		91.57	
ᅟ							
		18	Tumour	257	249.8 (40)	94.74	95.11 (0.54)
		27	Tumour	290.2		95.15	
		41	Tumour	258.3		95.85	
		57	Tumour	193.9		94.69	
ᅟ							
Replication Cohort	WASH U (Illumina All Exon 65 MB, Illumina HiSeq 2000, NovoAlign 2.07.13)	5	Proband	86.9	86.9	82.70	82.70
	BGI (Agilent SureSelect V4, Illumina HiSeq 2000, BWA 0.5.9)	20	Proband	27.6	28.1 (1.7)	50.41	50.95 (1.79)
		21	Proband	31.7		54.78	
		23	Proband	27.8		49.96	
		24	Proband	27.2		50.70	
		26	Proband	27.8		51.12	
		28	Proband	28		50.53	
		39	Proband	26.4		49.18	
	Perkin Elmer Corp (Agilent SureSelect Human All Exon 38 MB, Illumina HiSeq 2000, Bowtie 0.12.7)	A032	Proband	76.6	76.6	65.96	65.96

^a^ACHRI: Alberta Children’s Hospital Research Institute, Calgary, Canada; WASH U: Washington University, St. Louis, USA; BGI: Beijing Genome Institute, Beijing, China

### Structural variation analysis

Bellerophon v1.03 [23] was used with the default settings to search for chromosomal translocation and inter-chromosomal insertions located inside exons in all 16 ROHHAD proband exomes (the 7 discovery cohort trio probands and the 9 replication cohort exomes) and 4 tumour samples. FishingCNV v2.1 [24] was used to search for genomic copy number variants (CNVs) in the seven trio ROHHAD probands within the discovery cohort. Exomes from 22 healthy individuals, sequenced with the same enrichment kit (Agilent SureSelect capture kit V5 + UTRs), were used as controls. We used Benjamini-Hochberg multiple testing correction to detect CNVs with a p-value < 0.05.

### Variant validation and mutation analysis by sanger sequencing

The NCBI Primer Blast tool [25] was used to design site-specific primers to amplify genomic regions of interest. The amplicons were purified using the E.Z.N.A. Cycle Pure spin purification protocol (Omega Biotek) and then sequenced using the fluorescent dideoxy terminator method (Sanger method). Amplicon sequences were compared to the reference genome (GRCh37) using Mutation Surveyor (SoftGenetics) in order to identify variant positions within the exons or UTRs. All *de novo* candidate variants identified by DeNovoGear [15] with a posterior probability of 0.9 or greater were validated in this way, as were select candidate variants identified in the singleton (autosomal dominant and autosomal recessive) analyses. Selected candidate genes identified in the discovery cohort of 7 ROHHAD probands underwent mutation analysis by Sanger sequencing in 19 additional ROHHAD patients.

### Mutation analysis by exome sequencing

WES was obtained for nine members of the ROHHAD replication cohort, and was used to search for candidate variants in the genes identified as potential candidates through the analysis of the trio exomes (i.e., genes carrying *de novo* or compound heterozygous variants in one of the seven trios; or heterozygous, rare, protein-altering variants in three of the seven trio probands). Exome captures were completed using the Agilent SureSelect capture kit, 38 MB or V4, or the Illumina All Exon 65 MB kit. Massively parallel sequencing was performed on either a SOLiD or Illumina platform, at one of three institutions, and sequences were aligned to the human reference genome (GRCh37) using BWA 0.5.9 [26], Lifescope Genomic Analysis Software 2.5 (Life Technologies), NovoAlign 2.07.13 (Novocroft), or Bowtie 0.12.7 [27] (see Table 2). Variants were called using the GATK Haplotype Caller (Version 3.3) [16] and annotated for filtration and prioritization using ANNOVAR [17]. The coverage of these nine exomes was not sufficient for a robust whole exome analysis (Table 2), but provided a reasonable data set in which to perform an exploratory mutation analysis (Additional file 1 shows the low-coverage proportions for each candidate gene that underwent mutation analysis in these replication exomes).

## Results

### Cohort characteristics

A total of 35 ROHHAD patients in whom the clinical diagnosis was confirmed were included in this cohort for genetic investigation (Table 1). Specifically, 7 of these patients were included in the initial, trio-based, exome sequencing analysis (discovery cohort), while 28 other ROHHAD patients were included in the secondary exome and Sanger sequencing analysis (replication cohort). In addition, neural crest tumours from four ROHHAD patients (all of whom were trio probands) were analyzed. Blood DNA from 14 parents of the probands (7 trios) and from the monozygotic twin of one ROHHAD proband (discordant for the ROHHAD phenotype) was also included in our analysis. Our cohort represents a highly homogeneous group of ROHHAD patients, and all of the 35 patients included in the study showed rapid-onset obesity, hypothalamic dysfunction, hypoventilation, and autonomic dysregulation; all required artificial ventilation; and 15 (43 %) developed tumours of neural crest origin. Copy number variation (CNV) analysis by array comparative genomic hybridization (CGH), using the Nimblegen 720 k platform, in 26 of the 35 ROHHAD patients did not identify any ROHHAD-specific CNVs. Table 1 reveals a slight gender bias within our cohort (21 females, 14 males), however in our extensive experience studying ROHHAD we have not identified any specific gender prevalence, and careful review of ROHHAD referrals subsequent to this study identified equal distribution of females and males in our broader ROHHAD cohort.

### Exome sequencing

WES was completed on a total of 31 individuals including 16 ROHHAD patients (7 as part of trios in the discovery cohort and 9 in the replication cohort), 15 unaffected relatives (parents and one monozygotic twin), and tumour samples from four of the ROHHAD probands (Table 2).

#### De novo inheritance model - trio analysis

To identify *de novo* variants, we sequenced the exomes of seven ROHHAD trios. In this set of exomes, we achieved 130-fold mean coverage (after mapping and removal of PCR duplicate reads) with an average of 91.8 % +/− 0.81 % (SD) of bases covered at least 20-fold (Table 2). Using DeNovoGear [15] to analyze the trios, 13 candidate *de novo* variants were identified and validated by Sanger analysis (Table 3). Each patient carried between zero and three exonic or UTR *de novo* variants (average 1.86 per exome; 0.71 amino acid altering per exome), a value that is consistent with previous findings [28] and the expected mutation rate [29]. However, no two of these seven ROHHAD patients had *de novo* variants in the same gene.

**Table 3:** *De novo* variants observed in the exomes of seven ROHHAD cases (discovery cohort)

Proband ID	Gene	Selected transcript and variant effect	Variant type	CADD [21] (Phred scaled)	PolyPhen-2 [22] prediction (probability)	Genomic position (GRCh37)
Patient 18	*CD5*	NM_014207:c.1406A > G:p.E469G	Non-synonymous	21.1	Deleterious (1.000)	chr11:60893229
Patient 25	*CD36*	NM_001127444:c.1399A > G:p.R467G	Non-synonymous	19.84	Neutral (0.006)	chr7:80303443
	*C17orf53*	NM_024032:c.1046 T > C:p.I349T	Non-synonymous	5.791	Neutral (0.085)	chr17:42226217
	*NEK7*	NM_133494:c.*2404A > G	3′UTR	12.83	N/A	chr1:198291053
Patient 27	*MAPKAPK5*	NM_139078:c.664 T > A:p.C222S	Non-synonymous	22.6	Deleterious (1.000)	chr12:112321388
	*PPP1R16B*	NM_015568:c.477C > A:p.D159E	Non-synonymous	29.7	Deleterious (0.999)	chr20:37529233
	*TAF1*	NM_004606:c.4356C > T:p.R1452=	Synonymous	14.37	N/A	chrX:70627913
Patient 41	*PDE11A*	NM_001077358:c.783C > T:p.D261=	Synonymous	11.94	N/A	chr2:178592832
	*FAM155B*	NM_015686:c.*652G > A	3'UTR	1.311	N/A	chrX:68750448
Patient 42	*PLCXD3*	NM_001005473:c.*9191 T > C	3'UTR	4.216	N/A	chr5:41311356
Patient 45	*CCT4*	NM_006430:c.*613G > A	3'UTR	4.916	N/A	chr2:62095797
	*WDFY4*	NM_020945:c.8577G > T:p.T2859=	Synonymous	1.393	N/A	chr10:50174711
	*FAM199X*	NM_207318:c.828C > T:p.S276=	Synonymous	15.08	N/A	chrX:103432819

All variants are heterozygous

"*5" indicates the position 5 nucleotides 3' of the translation stop codon

In a second iteration of the genetic analysis, the 7 ROHHAD trio probands plus 9 additional ROHHAD exomes were then searched for other candidate variants (not necessarily *de novo*) in the 13 genes that had been flagged as containing *de novo* variants. Four genes were identified (*C17ORF53*, *PDE11A*, *WDFY4,* and *FAM199X*) that contained *de novo* variants in one patient each, and one or more additional candidate variants (novel or rare [MAF < 0.005] exonic, UTR, or splice site [within 2 bp of an exon] variants not within a segmental duplication) in other exomes (Table 4). From these four genes, *C17ORF53* was selected for mutation analysis in the replication cohort. It was selected because both of the candidate variants identified among the 16 patient exomes were amino acid altering, and neither was known to be inherited (one is known to be *de novo*; there was no inheritance information for the other). Although four different rare variants were identified among five patients in *PDE11A,* it was not selected for mutation analysis because in two cases, the variant was inherited from clinically unaffected parents, while another variant resulted in a synonymous change. *WDFY4* and *FAM199X* were each mutated in two individuals, but they were not considered for mutation analysis as, in both cases, one variant was shown to be inherited while the other variant resulted in a synonymous change.

**Table 4:** Results of extended *de novo* analysis^a^ and *C17ORF53* mutation analysis

Gene	Genomic position (GRCh37)	Variant type	Selected transcript and variant effect	MAF (Minor allele count/total allele count: 1000 genomes project; EVS; ExAC)^b^	CADD [21] (Phred scaled)	PolyPhen-2 [22] (probability)	Patient	Inheritance
*PDE11A*	chr2:178592832	Synonymous	NM_001077196: c.525C>T:p.D175D	Not found; Not Found; 0.000016 (2/122254)	11.94	N/A	Patient 41	*De novo*
	chr2:178528608	Non-synonymous	NM_001077196: c.1300A>G:p.M434V	0.0027 (6/2178); Not found; 0.00032 (39/122690)	18.54	Neutral (0.027)	Patient 24	Unknown
							Patient 18	Inherited (and present in MZ twin)
	chr2:178937010	Non-synonymous	NM_016953: c.155G>C:p.R52T	0.0005 (1/2178); 0.0025 (33/13006); 0.0016 (190/118894)	2.494	Neutral (0.016)	Patient 5	Unknown
	chr2:178936994	Frameshift (1 bp del)	NM_016953: c.171del:p.G57fs	Not found; Not found; Not found	22.6	N/A	Patient 42	Inherited
*C17ORF53* ^*c*^	chr17:42226217	Non-synonymous	NM_001171251: c.1046T>C:p.I349T	Not found; Not found; Not found	5.791	Neutral (0.085)	Patient 25	*De novo*
	chr17:42235240	Non-synonymous	NM_001171251: c.1810G>A:p.E604K	Not found; Not found; 0.000043 (5/117236)	7.093	Neutral (0.494)	Patient 5	Unknown
	**chr17:44162274**	**3′ UTR**	NM_001171251: c.*342C > G	**Not found; Not found; Not found**	**6.536**	**N/A**	**Patient 11**	**Unknown**
*WDFY4*	chr10:50174711	Synonymous	NM_020945: c.8577G > T:p.T2859=	Not found; Not found; Not found	1.393	N/A	Patient 45	*De novo*
	chr10:50186393	Non-synonymous	NM_020945: c.9331C > T:p.R3111W	0.0005 (1/2178); 0.00088 (4/4566); 0.00074 (15/20336)	6.501	Deleterious (0.66)	Patient 42	Inherited
*FAM199X*	chrX:103432819	Synonymous	NM_207318: c.828C > T:p.S276=	Not found; Not found; 0.000024 (3/122866)	15.08	N/A	Patient 45	*De novo*
	chrX:103435332	3'UTR	NM_207318: c.*876C > G	Not found; Not found; Not found	3.885	N/A	Patient 18	Inherited (and present in MZ twin)

^a^All 16 ROHHAD exomes were searched for candidate variants (as described in methods: novel or rare (MAF < 0.005) exonic, UTR, or splice site (within 2 bp of an exon) variants not within a segmental duplication) within the 13 genes identified as containing *de novo* variants in one ROHHAD proband

^b^1000 Genomes Project (http://www.1000genomes.org); EVS = Exome Variant Server (http://evs.gs.washington.edu/EVS/); ExAC = Exome Aggregation Consortium (http://exac.broadinstitute.org)

^c^ Of these four genes, *C17ORF53* was selected for mutation analysis in an additional 19 ROHHAD patients. One additional sequence variant was identified through this analysis, and is represented in the bolded row

#### Somatic mutation model (tumour and MZ twin analysis)

It is possible that *de novo* mutations giving rise to ROHHAD may be so severe that they are normally incompatible with life. Such a mechanism has been hypothesized for mutations in *AKT1*, which cause the Proteus syndrome, but have only been found in a mosaic state and never in all somatic cells [9]. Similarly, surviving patients with ROHHAD may be mosaic for the disease-causing mutation, carried in only a subset of their cells and tissues. If this model were applicable, the subset of ROHHAD patients with neural crest tumours would be the most likely to have an identifiable mutation, and the tumours themselves would be the most likely tissue to carry the causative mutation. To test this somatic mutation hypothesis, we sequenced the exomes of tumours from four of the seven patients that were included in the trio analysis.

 We assumed that the tumour samples would contain a variable amount of normal non-tumourous tissue. Because this mosaic state would reduce our ability to detect rare mutations, we increased the sequencing coverage to approximately double that obtained from the trios. This achieved a mean coverage of 260-fold (after mapping and removal of PCR duplicate reads) with an average of 95.1 % +/− 0.54 % (SD) of targeted bases covered at least 20-fold (Table 2). Since each analyzed tumour sample came from a ROHHAD case in a trio, DeNovoGear [15] was used to compare the tumour samples to the parental samples. Any *de novo* variants identified in a tumour were then compared to the *de novo* variants identified in that patient’s genomic DNA to single out any *de novo* variants unique to the tumour. All 13 of the *de novo* variants identified in the trio analysis were also present in the corresponding tumours; however, no additional tumour-only variants were detected.

Our cohort also contains a pair of monozygotic (MZ) twins discordant for the ROHHAD phenotype [30]. To evaluate the possibility of somatic mosaicism for a causative mutation in the affected twin, we used DeNovoGear to compare the variant profile between the twins, but did not identify any sequence variants in the affected twin’s exome that were not also present in the unaffected twin.

#### Autosomal recessive model

To identify potential recessive candidates, the trios were used to identify genes containing compound heterozygous inherited rare coding variants (or homozygous, as long as each parent was carrying the variant). We identified between one and four compound heterozygous candidate genes in each patient. These are genes that contained two rare variants inherited in trans (i.e., one from each parent) (Table 5). No individual compound heterozygous candidate was seen in more than one individual, none of the nine replication exomes were found to contain two sequence variants in any of these genes, and none of these candidates have a known function that would strongly suggest an association with ROHHAD. Thus, we have not pursued any of them further at this time. We acknowledge that, in light of the fact that ROHHAD may be highly genetically heterogeneous, one or more of these genes may indeed be disease-causing in an individual proband – but further studies would be required in order to gain additional evidence in support of any particular gene.

**Table 5:** Compound heterozygous variants observed in exomes of seven ROHHAD cases (discovery cohort)

Proband ID	Gene	Variant type	Selected transcript and variant effect	Inherited from	Polyphen-2 [22] Prediction (Probability)	CADD [21] Score (Phred-scaled	MAF (1000 genomes Project; EVS; ExAC)^a^	Total reads (% of reads supporting variant Allele)
18	*MAST4*	Non-synonymous	MAST4:NM_001297651:	Mother	Deleterious (0.998)	21.4	Not found; Not found; 0.00004466	198 (40 %)
			exon1:c.34C>T:p.L12F					
		Non-synonymous	MAST4:NM_001297651:	Father	Deleterious (1.000)	20.9	Not found; Not found; 0.0002	92 (35 %)
			exon26:c.4690C>T:p.L1564F					
	*OTOG*	Non-synonymous	OTOG:NM_001277269:	Mother	Deleterious (1.000)	20.1	Not found; Not found; Not found	31 (32 %)
			exon47:c.7907G>A:p.R2636H					
		synonymous	OTOG:NM_001277269:	Father	N/A	9.71	Not found; Not found; Not found	63 (48 %)
			exon37:c.6381C>T:p.H2127H					
25	*DNAH11*	synonymous	DNAH11:NM_001277115:	Father	N/A	0.005	0.00159744; 0.0032; 0.003	177 (36 %)
			exon16:c.3237T>C:p.L1079L					
		synonymous	DNAH11:NM_001277115:	Mother	N/A	0.002	0.000199681; 0.0027; 0.0024	53 (49 %)
			exon57:c.9468T>C:p.D3156D					
	*DMXL2*	Non-synonymous	DMXL2:NM_001174117:	Mother	Neutral (0.029)	14.65	Not found; Not found; 0.0001	34 (32 %)
			exon39:c.6727G>C:p.V2243L					
		synonymous	DMXL2:NM_001174116:	Father	N/A	0.646	Not found; 0.0004; 0.0005	126 (44 %)
			exon8:c.861C>T:p.T287T					
27	*SACS*	Non-synonymous	SACS:NM_001278055:	Mother	Deleterious (0.999)	21.8	0.000798722; 0.0035; 0.0028	87 (40 %)
			exon8:c.7898T>G:p.F2633C					
		Non-synonymous	SACS:NM_001278055:	Father	Deleterious (0.996)	16.19	Not found; Not found; Not found	101 (45 %)
			exon8:c.6009G>T:p.Q2003H					
	*ZNF44*	Non-synonymous	ZNF44:NM_016264:	Father	Deleterious (1.000)	16.13	Not found; Not found; 0.0002	123 (45 %)
			exon4:c.923C>T:p.P308L					
		frameshift deletion	ZNF44:NM_016264:	Mother	N/A	23.3	Not found; Not found; 0.00008261	145 (43 %)
			exon4:c.562_563del:p.M188fs					
41	*C15orf39*	synonymous	C15orf39:NM_015492:	Father	N/A	6.427	0.00139776; 0.0032; 0.0021	157 (38 %)
			exon2:c.702C>T:p.Y234Y					
		nonframeshift deletion	C15orf39:NM_015492:exon2:	Mother	N/A	33	Not found; 0.0026; 0.0001	56 (30 %)
			c.2210_2221del:p.737_741del					
	*CD300LF*	Non-synonymous	CD300LF:NM_001289083:	Mother	Neutral (0.278)	0.265	Not found; Not found; 0.0000249	37 (43 %)
			exon5:c.542T>C:p.I181T					
		Non-synonymous	CD300LF:NM_001289083:	Father	Deleterious (0.746)	13.19	Not found; Not found; 0.000033	118 (43 %)
			exon2:c.336A>C:p.K112N					
	*ZFHX4*	synonymous	ZFHX4:NM_024721:	Mother	N/A	14.05	0.000199681; 0.0002; 0.000075	113 (36 %)
			exon3:c.3075G>A:p.A1025A					
		Non-synonymous	ZFHX4:NM_024721:	Father	Neutral (0.000)	1.699	Not found; Not found; Not found	171 (39 %)
			exon10:c.7262C>T:p.P2421L					
		synonymous	ZFHX4:NM_024721:	Mother	N/A	3.092	0.00179712; 0.0019; 0.0025	125 (39 %)
			exon11:c.9960A>G:p.Q3320Q					
42	*DST*	Non-synonymous	DST:NM_015548:	Mother	Deleterious (1.000)	15.35	Not found; Not found; Not found	197 (43 %)
			exon42:c.8467T>C:p.C2823R					
		Non-synonymous	DST:NM_015548:	Father	Neutral (0.144)	12.02	Not found; Not found; Not found	127 (39 %)
			exon14:c.2124A>G:p.I708M					
	*EYS*	Non-synonymous	EYS:NM_001142800:	Mother	Deleterious (1.000)	14.25	Not found; Not found; Not found	183 (42 %)
			exon40:c.7792G>A:p.G2598S					
		Non-synonymous	EYS:NM_001142800:	Father	Neutral (0.031)	11.83	Not found; Not found; 0.000008239	189 (38 %)
			exon4:c.455T>C:p.M152T					
45	*SPEG*	Non-synonymous	SPEG:NM_005876:	Mother	Neutral (0.235)	15.47	Not found; Not found; Not found	25 (24 %)
			exon17:c.4181A>G:p.D1394G					
		Non-synonymous	SPEG:NM_005876:	Father	Neutral (0.244)	4.916	Not found; 0.0003; 0.0003	17 (59 %)
			exon30:c.6854C>T:p.P2285L					
57	*ALS2CR11*	Non-synonymous	ALS2CR11:NM_001168221:	Father	Neutral (0.008)	6.899	Not found; Not found; Not found	30 (47 %)
			exon15:c.4114G>A:p.D1372N					
		Non-synonymous	ALS2CR11:NM_001168221:	Mother	Neutral (0.125)	16.49	0.000199681; Not found; Not found	40 (55 %)
			exon15:c.2216A>G:p.K739R					
	*SYNE1*	Non-synonymous	SYNE1:NM_033071:	Mother	Deleterious (0.982)	25.8	0.000199681; 0.0006; 0.0005	214 (40 %)
			exon142:c.25607A>C:p.D8536A					
		Non-synonymous	SYNE1:NM_033071:	Father	Deleterious (0.999)	34	Not found; 0.0002; 0.000008238	93 (51 %)
			exon102:c.19015G>A:p.E6339K					

^a^1000 Genomes Project (http://www.1000genomes.org); EVS = Exome Variant Server (http://evs.gs.washington.edu/EVS/); ExAC = Exome Aggregation Consortium (http://exac.broadinstitute.org)

#### Autosomal dominant model

Though a familial dominant inheritance model is not consistent with the sporadic occurrence of this disease, we did search the 7 ROHHAD patient exomes for any genes that contained rare protein-altering (i.e., missense, nonsense, splice-site and indel) variants in all or a large subset of the patients after censoring the *de novo* or inherited status. After further filtering by visual inspection in the Integrative Genomics Viewer (IGV; [31, 32]), multiple variant calls were removed as probable false positives (e.g. eliminating rare variants on reads that hosted multiple other rare variants – we assumed that these reads were poorly mapped). At this stage, we identified no genes with candidate variants in the same gene among 4 or more of the 7 patients. We did identify four genes (*FRAS1, RELN, RIF1, POLE*) that each had different rare, protein-altering variants among 3 of the 7 patients (Table 6). We searched the 9 exomes from the replication cohort for rare protein-altering variants in these four genes and identified only one additional patient with a candidate variant in *RIF1* (Table 6). All four of the *RIF1* variants were validated by Sanger sequencing. All four RIF1 variants as well as all variants in the three genes in which three of 16 probands have candidate variants (*FRAS1, RELN, POLE*), were inherited from unaffected parents, with both maternal and paternal transmission (Table 6).

**Table 6:** Genes with candidate variants observed in three of seven ROHHAD cases

Gene	Proband ID	Variant type	Selected transcript and variant effect	Polyphen-2 [22] Prediction (Probability)	CADD [21] score (Phred-scaled)	MAF (1000 genomes project; EVS; ExAC)^a^	Inherited from	No. of reads supporting reference call, No. of reads supporting variant call
*FRAS1*	41	Non-synonymous	NM_001166133: exon27:c.3500G>A:p.R1167H	Neutral (0)	0.01	Not Found; 0.000083; 0.000058	Mother	64,50
	42	Non-synonymous	NM_001166133: exon29:c.3963G>C:p.K1321N	Neutral (0.076)	9.266	Not Found; 0.000082; 0.0000516	Mother	96,57
	57	Non-synonymous	NM_001166133: exon38:c.5003A>G:p.N1668S	Deleterious (0.996)	15.68	Not Found; Not Found; Not Found	Father	86,72
*RELN*	57	Non-synonymous	NM_005045: exon54:c.8798C>T:p.T2933I	Neutral (0.124)	16.6	0.000599042; 0.0002; 0.0002	Father	110,78
	27	Non-synonymous	NM_005045: exon51:c.8254G>A:p.G2752S	Neutral (0.405)	23.3	Not Found; Not Found; Not Found	Mother	12,14
	45	Non-synonymous	NM_005045: exon26:c.3651C>G:p.I1217M	Deleterious (0.812)	19.32	0.000998403; 0.0034; 0.0027	Father	45,18
*RIF1*	18	Non-synonymous	NM_018151: exon30:c.6314T>C:p.M2105T	Neutral (0.002)	9.267	Not Found; Not Found; 0.0000165	Mother	59,50
	41						Father	37,41
	42	Splicing	NM_018151: exon32:c.6825 + 2 T > C	N/A	23.9	Not Found; Not Found; Not Found	Father	48,34
	**23**	**Non-synonymous**	**NM_001177665: exon4:c.346C>T:p.R116C**	**Neutral (0.09)**	**14.47**	**Not Found; 0.0004; 0.0003**	**Unknown**	**84,90**
*POLE*	27	Non-synonymous	NM_006231: exon43:c.5965G>A:p.A1989T	Neutral (0.043)	15.8	Not Found; Not Found; Not Found	Mother	37,20
	41	Non-synonymous	NM_006231: exon41:c.5659G>A:p.V1887M	Neutral (0.305)	10.07	Not Found; 0.0006; 0.0005	Mother	43,36
	42	Non-synonymous	NM_006231: exon13:c.1288G>A:p.A430T	Deleterious (0.977)	24.5	0.000998403; 0.000077; 0.0007	Father	23,21

Bolded row represents variant identified in replication exomes

^a^1000 Genomes Project (http://www.1000genomes.org); EVS = Exome Variant Server (http://evs.gs.washington.edu/EVS/); ExAC = Exome Aggregation Consortium (http://exac.broadinstitute.org)

Additionally, 187 genes were identified with potential variants in 2 of the 7 discovery cohort patients (Additional file 2), but these have not been inspected in IGV, and a portion of them are likely false positive calls. While it remains possible that sequence variants in one or more of these genes are causative of ROHHAD, with such a large number of candidate genes, and without inheritance information to support any particular one, it presently remains difficult to obtain enough genetic evidence to identify one as a causative gene. In one attempt to narrow down this list, we looked only at loss-of-function (LOF) variants, but did not identify any gene with LOF variants in more than one proband.

### Structural variation analysis

In addition to identifying SNVs and small indels (as described in the previous sections), we also analyzed the ROHHAD exome data (including the tumour samples) for larger variations such as chromosomal translocations and inter chromosomal insertions and CNVs. No CNVs and no exonic translocations or insertions were detected in any of the samples. However, with exome data alone, we cannot rule out the possibility of fusion events in intronic or intergenic regions.

### Mutation analysis

We performed mutation analysis (by Sanger sequencing) of two candidate genes (*C17ORF53* and *MAPKAPK5*) in 19 additional ROHHAD patients.

#### C17ORF53

In total, we identified three novel or rare (MAF < 0.005) variants in a search of the exons and UTRs of the *C17ORF53* gene among 35 ROHHAD patients (16 by exome sequencing and 19 by Sanger sequencing) (Table 4). Two were non-synonymous variants, and one was in the 3’UTR. We do not have genetic samples from the parents of two of the patients carrying sequence variants in this gene, and so are unable to determine whether or not they are *de novo* variants. Since very little is known about the protein encoded by this open reading frame, we do not have information to model the effect these sequence variants would have. Further investigation into the protein (when and where it is expressed, and what its function is) and the effect of these variants would be required to further implicate them in ROHHAD.

#### MAPKAPK5

Improvement in a subset of phenotypic features in Patient 27 was observed shortly after a regimen of caffeine treatment (100 mg BID) was initiated [33]. A study found that transcript levels of *MAPKAPK5* (in which Patient 27 is carrying a *de novo* missense variant; Table 3) were transiently increased in response to forskolin treatment (which, like caffeine, elevates cAMP) [34], leading us to hypothesize that *MAPKAPK5* might be involved in this patient’s ROHHAD phenotype. However, mutation analysis in our ROHHAD cohort did not identify any additional novel or rare exonic, splice site, or UTR sequence variants in *MAPKAPK5*.

## Discussion

Our hypothesis that ROHHAD might be caused by germline *de novo* mutations or by mosaicism for somatic mutation(s) in one or more genes was tested using seven trios, including neural crest tumours of four of these patients and the unaffected MZ twin of one (Table 2), as well as an additional 28 singleton ROHHAD patients used for mutation analysis of candidate genes. Despite the homogeneity of this ROHHAD cohort, and the depth of coverage established in our exome sequencing, we did not identify any two patients who had *de novo* variants in the same gene, any *de novo* coding variants unique to the tumours, or any variants discordant between the affected and unaffected MZ twins. We also did not identify any structural variations in the exomes. While the sporadic occurrence of ROHHAD suggests a *de novo* inheritance model, we were also able to use the exome sequencing data to assess autosomal recessive and autosomal dominant inheritance models. Neither model revealed a major gene that would explain ROHHAD in our cohort. One or more of the genes containing a) *de novo* variants (Table 3, Table 4), b) inherited compound heterozygous variants (Table 5), or c) inherited heterozygous variants in 3 or 4 patients (Table 6), may be causative of ROHHAD in some cases, but further genetic and/or functional evidence is required before these variants could be considered truly pathogenic for the ROHHAD phenotype. In particular, it is difficult to determine the significance of the *RIF1* finding (that 4 of 16 patients have inherited a rare protein-altering variant in this gene). The gene is large (41 exons encoding 2472 amino acids), and harbours a substantial number of variants (2492 variants in the ExAC database [20], 937 of which are rare protein-altering variants, and 20 of which are loss-of-function (LOF) variants [i.e., nonsense, frameshift, or canonical splice site mutations]). Thus, additional evidence - genetic (i.e., identifying additional ROHHAD patients with *RIF1* mutations) or functional (i.e., discovering a *RIF1* function that could explain the ROHHHAD phenotype) – would be required to prove that this is more than a random chance occurrence. An *in silico* search using Ingenuity Pathway Analysis (Qiagen) for functional connections among these 31 genes did not identify any potential links between different candidate genes from more than two patients.

Previous studies postulated eight genes (*NDN*, *ASCL1*, *PHOX2B*, *NTRK2*, *BDNF*, *HTR1A*, *OTP*, and *ADCYAP1*) as candidate ROHHAD genes, based on their roles in the development or function of the systems (hypothalamic, autonomic, and neuroendocrine) known to be deficient in ROHHAD, but failed to identify any disease-associated variants in these genes among their cohorts [2, 4, 13]. Likewise, we did not identify any candidate variants in any of these eight genes in our 7 ROHHAD patient exomes (for each gene, at least 96 % of the coding bases were covered at least 20-fold with the exception of OTP, for which one of three exons was not effectively captured), confirming that none of these eight genes is a major ROHHAD gene. A recent study described a novel nonsense mutation in the Smith-Magenis gene, *RAI1,* in a patient previously diagnosed with ROHHAD (though the authors note that deeper evaluation revealed his phenotype was not consistent with ROHHAD or Smith-Magenis syndrome) [35]. We did not identify any candidate variants in *RAI1* in our 7 ROHHAD patient exomes, despite the fact that >96 % of coding bases were covered at least 20-fold, suggesting that mutations in this gene are not characteristic of ROHHAD.

Although the present analysis did not uncover a causative gene for ROHHAD, it does not completely rule out the possibility that ROHHAD is caused by *de novo* coding mutations. There are several aspects of the disease that may lead our analysis, as currently designed, to miss a *de novo* coding answer, even if one exists. For one, the possibility that somatic mosaicism represents the major mechanism for ROHHAD complicates our analysis in that we may not have sampled the major tissue(s) carrying the ROHHAD-causing mutations, such as the brainstem or hypothalamic nuclei. Our use of the patients’ neuroendocrine tumours represents one approach to addressing this challenge, because these tissues would be expected to contain the causative ROHHAD genetic lesion if one exists. The fact that we did not identify any coding mutations distinct to the tumours suggests that the genetic lesion(s) we are looking for may be rare and highly specific (such as regulatory mutations or fusion genes) or are located outside the exonic region. Secondly, there is the possibility that ROHHAD is characterized by a high level of genetic heterogeneity, whereby a discovery set of seven probands is not sufficiently large to contain more than one patient with a mutation in any single ROHHAD gene. However, we think that this is unlikely, as we did extend our exome analysis to include a total of 16 ROHHAD patients, and our entire study cohort included a total of 35 ROHHAD patients, suggesting that exome sequencing of additional trios or individuals is not likely to lead to an answer for the underlying genetics of ROHHAD. Finally, some limitations remain as to what can be discovered, even within the coding region, by WES. The mutations causing ROHHAD may be of a nature that is difficult to detect by WES, such as triplet repeat expansion. Alternatively, causative mutations might have occurred in parts of genes poorly enriched by our capture kit, and thus not covered by our sequencing. A list of those genes reveals several genes with known functions that could potentially be linked to ROHHAD; those will require a more extensive follow up sequencing project.

Given the many recent successes of WES in identifying disease-causing mutations, our study represents a major effort toward discovering the cause of ROHHAD. Though no coding pathogenic mutations for this disease were identified, our experimental design was sufficiently robust for us to conclude that exome sequencing of trios and tumours is not likely to be an efficient means of identifying the cause of ROHHAD. A reasonable next step would be whole genome sequencing to look for *de novo* mutations beyond the coding exome, that may disrupt promoter regions, transcription factor binding sites, microRNA genes or binding sites, or other important non-coding regions.

It is also important to consider alternative hypotheses wherein ROHHAD may not have a genetic etiology. Older literature has suggested that ROHHAD may be a paraneoplastic condition, resulting from pathophysiology triggered by a specific subset of neuroendocrine tumours [6, 36, 37]. Nevertheless, the fact that many patients with ROHHAD do not have neuroendocrine tumours suggests that the paraneoplastic hypothesis cannot be the single unifying model. Similarly, the fact that patients whose tumours were removed do not experience remission of other symptoms, and the ROHHAD phenotype continues to evolve with advanced hypoventilation and autonomic dysregulation, makes this model less attractive as an explanation. An autoimmune etiology for ROHHAD has also been postulated [6, 38, 39] though it is not clear that all the study subjects actually had ROHHAD, or that the intervention modified the phenotype. No technology yet available can rule out an autoimmune etiology for a particular disease, though autoimmune-mediated inflammation cannot explain a predisposition to neoplasia.

## Conclusion

Using a comprehensive, well-designed and carefully executed exome sequencing study in an expertly-phenotyped cohort of 35 ROHHAD patients, we were not able to identify *de novo* pathogenic mutations causing ROHHAD. Our findings suggest that clinical exome sequencing is unlikely to be a useful diagnostic test in patients with true ROHHAD. However, because ROHHAD is a devastating disease that is characterized by a high incidence of cardiorespiratory arrest in childhood and by the need for artificial ventilation for life-support, it remains imperative to refine the search for genetic risk factors for ROHHAD, as well as to investigate other possible mechanisms of disease.

## Additional files

Additional file 1: 
**Low-coverage proportions of candidate genes in replication exomes.** (XLSX 43 kb) Additional file 2: 
**Genes carrying putative candidate mutations in 2 of 7 ROHHAD probands in the discovery cohort.** (XLSX 44 kb)

## References

[1] Fishman LS, Samson JH, Sperling DR (1965). Primary alveolar hypoventilation syndrome (Ondine’s Curse). Am J Dis Child.

[2] Ize-Ludlow D (2007). Rapid-onset obesity with hypothalamic dysfunction, hypoventilation, and autonomic dysregulation presenting in childhood. Pediatrics.

[3] Bougneres P (2008). Endocrine manifestations of the rapid-onset obesity with hypoventilation, hypothalamic, autonomic dysregulation, and neural tumor syndrome in childhood. J Clin Endocrinol Metab.

[4] De Pontual L (2008). Delineation of late onset hypoventilation associated with hypothalamic dysfunction syndrome. Pediatr Res.

[5] Onal H, Ersen A (2010). A case of late-onset central hypoventilation syndrome with hypothalamic dysfunction: through a new phenotype. Turk J Pediatr.

[6] Paz-Priel I, Cooke DW, Chen AR (2011). Cyclophosphamide for rapid-onset obesity, hypothalamic dysfunction, hypoventilation, and autonomic dysregulation syndrome. J Pediatr.

[7] Weese-Mayer DE (2010). An official Ats clinical policy statement: congenital central hypoventilation syndrome: genetic basis, diagnosis, and management. Am J Respir Crit Care Med.

[8] Douglas J (2003). Nsd1 mutations are the major cause of sotos syndrome and occur in some cases of weaver syndrome but are rare in other overgrowth phenotypes. Am J Hum Genet.

[9] Lindhurst MJ (2011). A mosaic activating mutation in Akt1 associated with the Proteus syndrome. N Engl J Med.

[10] Kurek KC (2012). Somatic mosaic activating mutations in Pik3ca Cause Cloves syndrome. Am J Hum Genet.

[11] Gibson WT (2012). Mutations in Ezh2 cause Weaver syndrome. Am J Hum Genet.

[12] Weksberg R (2003). Beckwith-Wiedemann syndrome demonstrates a role for epigenetic control of normal development. Hum Mol Genet.

[13] Rand CM (2011). Rapid-onset obesity with hypothalamic dysfunction, hypoventilation, and autonomic dysregulation: analysis of hypothalamic and autonomic candidate genes. Pediatr Res.

[14] Beaulieu CL (2014). Forge Canada consortium: outcomes of A 2-year national rare-disease gene-discovery project. Am J Hum Genet.

[15] Ramu A (2013). Denovogear: De Novo Indel and point mutation discovery and phasing. Nat Methods.

[16] Van Der Auwera, GA, et al. From Fastq Data To High Confidence Variant Calls: The Genome Analysis Toolkit Best Practices Pipeline. Current Protocols In Bioinformatics/Editoral Board, Andreas D. Baxevanis et al. 2013. 11(1110): 11 10 1–11 10 33. 10.1002/0471250953.bi1110s43 PMC4243306 25431634

[17] Wang K, Li M, Hakonarson H (2010). Annovar: functional annotation of genetic variants from high-throughput sequencing data. Nucleic Acids Res.

[18] Abecasis GR (2012). An integrated map of genetic variation from 1,092 human genomes. Nature.

[19] Exome Variant Server, Nhlbi Go Exome Sequencing Project (Esp), Seattle, Wa. December, 2014. Available From: Http://Evs.Gs.Washington.Edu/Evs/.

[20] Exome Aggregation Consortium (Exac), Cambridge, Ma. Available From: http://exac.broadinstitute.org.

[21] Kircher M (2014). A general framework for estimating the relative pathogenicity of human genetic variants. Nat Genet.

[22] Adzhubei IA (2010). A method and server for predicting damaging missense mutations. Nat Methods.

[23] Hayes M, Li J (2013). Bellerophon: a hybrid method for detecting interchromosomal rearrangements at base pair resolution using next-generation sequencing data. BMC Bioinformatics.

[24] Shi Y, Majewski J (2013). Fishingcnv: a graphical software package for detecting rare copy number variations in exome-sequencing data. Bioinformatics.

[25] Ye J (2012). Primer-blast: a tool to design target-specific primers for polymerase chain reaction. BMC Bioinformatics.

[26] Li H, Durbin R (2009). Fast and accurate short read alignment with burrows-wheeler transform. Bioinformatics.

[27] Langmead B (2009). Ultrafast and memory-efficient alignment of short Dna sequences to the human genome. Genome Biol.

[28] O’roak BJ (2011). Exome sequencing in sporadic autism spectrum disorders identifies severe de novo mutations. Nat Genet.

[29] Lynch M (2010). Rate, molecular spectrum, and consequences of human mutation. Proc Natl Acad Sci U S A.

[30] Patwari PP (2011). Monozygotic twins discordant for Rohhad phenotype. Pediatrics.

[31] Robinson JT (2011). Integrative genomics viewer. Nat Biotechnol.

[32] Thorvaldsdottir H, Robinson JT, Mesirov JP (2013). Integrative Genomics Viewer (Igv): high-performance genomics data visualization and exploration. Brief Bioinform.

[33] Gordon, SC, et al. The evolving phenotype in a patient with Rapid-Onset Obesity With Hypothalamic Dysfunction, Hypoventilation, And Autonomic Dysregulation (Rohhad) and response to caffeine treatment. Am J Respir Crit Care. 2015. Med(191): P. A5923, 2015 - Presented At American Thoracic Society International Conference, Denver, Co, May 2015.

[34] Gerits N (2009). The transcriptional regulation and cell-specific expression of the Mapk-activated protein kinase Mk5. Cell Mol Biol Lett.

[35] Thaker VV (2015). Whole exome sequencing identifies rai1 mutation in a morbidly obese child diagnosed with rohhad syndrome. J Clin Endocrinol Metab.

[36] Ouvrier R (1995). Idiopathic hypothalamic dysfunction: a paraneoplastic syndrome?. Lancet.

[37] Sirvent N (2003). Hypothalamic dysfunction associated with neuroblastoma: evidence for a new paraneoplastic syndrome?. Med Pediatr Oncol.

[38] Sartori S (2014). Intrathecal synthesis of oligoclonal bands in rapid-onset obesity with hypothalamic dysfunction, hypoventilation, and autonomic dysregulation syndrome: new evidence supporting immunological pathogenesis. J Child Neurol.

[39] Chow C (2015). Rapid-Onset Obesity with Hypothalamic Dysfunction, Hypoventilation, and Autonomic Dysregulation (Rohhad) syndrome may have a hypothalamus-periaqueductal gray localization. Pediatr Neurol.

